# The 10th Biennial Hatter Cardiovascular Institute workshop: cellular protection—evaluating new directions in the setting of myocardial infarction, ischaemic stroke, and cardio-oncology

**DOI:** 10.1007/s00395-018-0704-z

**Published:** 2018-10-11

**Authors:** Sean M. Davidson, Sapna Arjun, Maryna V. Basalay, Robert M. Bell, Daniel I. Bromage, Hans Erik Bøtker, Richard D. Carr, John Cunningham, Arjun K. Ghosh, Gerd Heusch, Borja Ibanez, Petra Kleinbongard, Sandrine Lecour, Helen Maddock, Michel Ovize, Malcolm Walker, Marlene Wiart, Derek M. Yellon

**Affiliations:** 1 The Hatter Cardiovascular Institute, Institute of Cardiovascular Science, University College London, 67 Chenies Mews, London, WC1E 6HX UK; 2 School of Cardiovascular Medicine and Sciences, King’s College London British Heart Foundation Centre of Excellence, James Black Centre, 125 Coldharbour Lane, London, SE5 9NU UK; 3 Department of Cardiology, Aarhus University Hospital, Palle Juul-Jensens Boulevard 99, 8200 Aarhus N, Denmark; 4 MSD A/S, Copenhagen, Denmark; 5 Centre for Nephrology, UCL Medical School, Royal Free Campus, Rowland Hill Street, London, NW3 2PF UK; 6 West German Heart and Vascular Center, Institute for Pathophysiology, University of Essen Medical School, Essen, Germany; 7 Centro Nacional de Investigaciones Cardiovasculares (CNIC), Madrid, Spain; 8 CIBER de Enfermedades CardioVasculares, Madrid, Spain; 9 IIS-Fundación Jiménez Díaz University Hospital, Madrid, Spain; 10 Cardioprotection Group, Hatter Institute for Cardiovascular Research in Africa, University of Cape Town, Cape Town, South Africa; 11 Centre for Sport, Exercise and Life Sciences, Faculty of Health and Life Sciences, Coventry University, Priory Street, Coventry, CV1 5FB UK; 12 INSERM U1060, CarMeN Laboratory, Université de Lyon and Service d’explorations Fonctionnelles Cardiovasculaires Groupement Hospitalier Est, 59 Boulevard Pinel, 69500 Bron, France; 13 CNRS, Lyon, France

**Keywords:** Anthracycline cardiotoxicity, Cardioprotection, Ischaemic stroke, Myocardial ischaemia, Neuroprotection, Reperfusion

## Abstract

Due to its poor capacity for regeneration, the heart is particularly sensitive to the loss of contractile cardiomyocytes. The onslaught of damage caused by ischaemia and reperfusion, occurring during an acute myocardial infarction and the subsequent reperfusion therapy, can wipe out upwards of a billion cardiomyocytes. A similar program of cell death can cause the irreversible loss of neurons in ischaemic stroke. Similar pathways of lethal cell injury can contribute to other pathologies such as left ventricular dysfunction and heart failure caused by cancer therapy. Consequently, strategies designed to protect the heart from lethal cell injury have the potential to be applicable across all three pathologies. The investigators meeting at the 10th Hatter Cardiovascular Institute workshop examined the parallels between ST-segment elevation myocardial infarction (STEMI), ischaemic stroke, and other pathologies that cause the loss of cardiomyocytes including cancer therapeutic cardiotoxicity. They examined the prospects for protection by remote ischaemic conditioning (RIC) in each scenario, and evaluated impasses and novel opportunities for cellular protection, with the future landscape for RIC in the clinical setting to be determined by the outcome of the large ERIC-PPCI/CONDI2 study. It was agreed that the way forward must include measures to improve experimental methodologies, such that they better reflect the clinical scenario and to judiciously select combinations of therapies targeting specific pathways of cellular death and injury.

## Introduction

Unlike some organs that readily regenerate following injury, the adult heart lacks meaningful quantities of endogenous stem cells able to regenerate cardiomyocytes [70]—when a cardiomyocyte is lost, it is gone forever. It is, therefore, imperative to preserve the ones we have. In most cases, the heart endures extraordinarily well, continuing to function for upwards of 70 years or more with exactly the same cardiomyocytes it started with. However, an ST-segment elevation myocardial infarction (STEMI) causes an onslaught of damage that can wipe out over a billion cardiomyocytes [77]. Of patients who reach the hospital and are treated with optimal therapy, > 10% will die within 1 year, and many of those who survive will go on to develop heart failure as a consequence of the initial infarct [53, 82]. Over the past 25 years, the creation of an emergency care infrastructure enabling rapid myocardial reperfusion has greatly improved clinical outcomes [90]. Unfortunately, in many countries, the reward available from further logistical improvements in the implementation of this intervention appears to have reached its practical limit. For example, in the recent SWEDE HEART study, despite an impressive decrease in the numbers of deaths following STEMI, made after the introduction of emergency coronary care and the implementation of reperfusion therapy, 1-year mortality has remained stubbornly high at ~ 15% [95].

Like the heart, the adult brain has extremely limited capacity to make new cells, and acute obstruction of a conduit artery causes the irreversible loss of cells—a typical ischaemic stroke causes the loss of ~ 1 billion neurons [87]. Stroke causes 9% of all deaths making it the second leading cause of death and one of the most costly and devastating clinical syndromes in the world [30]. Approximately 20% of strokes are caused by intracerebral haemorrhage, while the other ~ 80% are classified as ischaemic. With the discovery of thrombolysis, reperfusion therapy became an option for the treatment of ischaemic stroke. More recently, the introduction of mechanical thrombectomy has brought about a paradigm shift in the optimal management of ischaemic stroke patients, in particular those with large vessel occlusion who had had poor recanalization rates with thrombolysis [18]. Endovascular recanalization results in rapid restoration of blood flow to the ischaemic cerebrum with the promise of improving neurological salvage and functional outcome. The sequelae of reperfusion for stroke are similar to those seen during primary percutaneous coronary intervention for STEMI [58]. Importantly, in both the brain [35] and myocardium [58], early reperfusion is the only therapy that is proven to limit infarct size in patients. However, a substantial number of stroke patients who receive thrombolysis and/or thrombectomy in the acute phase never fully recover [35]. This highlights the need to develop new adjunctive neuroprotective treatment strategies alongside reperfusion therapy.

Another of the major killers worldwide is cancer, which affects more than one in three people in their lifetime [74]. Anthracyclines such as doxorubicin are highly effective and commonly used chemotherapeutic agents, but are restricted by dose-limiting cardiotoxicity [74]. Although the incidence of anthracycline-induced cardiomyopathy has declined with contemporary dosing regimens, a significant number of patients develop left ventricular dysfunction and heart failure. The exact proportion of patients affected is difficult to ascertain due primarily to methodological issues, but has been estimated to be in the range of 3–26% [14]. The cause of myocardial injury is multifarious, but is believed to include oxidative stress, inhibition of topoisomerase II β, mitochondrial dysfunction, and deficits in cardiomyocyte energy production, which lead to diffuse cardiomyocyte death [14, 37, 38, 74]. Although discussion focussed on anthracyclines, other types of cancer therapy such as HER2 inhibitors can cause similar cardiac injury leading to heart failure [24, 97].

In all three fields mentioned above, and discussed during this workshop, discoveries of protective agents that are effective in experimental studies have failed to translate well to clinical studies in patients. The reasons for this have been extensively discussed in debates that have progressed similarly in each of the research domains. In the field of cardioprotection, recommendations have been published including those deriving from previous Hatter Institute workshops [11, 12, 15, 43, 67]. In neuroprotection, the Stroke Treatment Academic Industry Roundtable (STAIR) guidelines defined similar standards for optimal experimental design [34], which have been improved upon over subsequent years [65, 66]. Recommendations have also been published for pre-clinical studies of chemotherapy-induced cardiotoxicity and associated assessment of early subclinical myocardial injury biomarkers such as microRNAs [72, 86]. A common theme in these guidelines is the apparent disconnect between overly simplistic experimental models using young, healthy animals, and the complex reality of the clinical scenario [11, 12, 15, 34, 43, 47, 49, 55, 67, 84].

In view of the above, a key question discussed at the workshop was whether there is any commonality between the mechanisms of cell death that occur in these three pathologies and, if so, whether this knowledge can inform the development of improved cytoprotective modalities that are able to improve clinical outcome in patients.

### The same but different

Superficially, at least, there are a number of obvious commonalities between STEMI and ischaemic stroke, which raise the interesting possibility that protective modalities successful in one scenario may also be effective in the other (Table 1). On the other hand, there are clearly also specific differences that may impede the blanket application of therapies across these scenarios (Table 1). While cardiomyocyte death is also integral to cardiotoxicity after cancer chemotherapy, its similarity to the cell death that occurs during ischaemia–reperfusion is more controversial and will be discussed later.

**Table 1:** Commonalities and differences between the typical patient in the setting of STEMI, ischaemic stroke and anthracycline chemotherapy, who may be amenable for cardioprotective or neuroprotective strategies

	Myocardial infarction	Ischaemic stroke	Cancer chemotherapy cardiotoxicity
Patient Identification	ECG + Biomarker	CT (computed tomography) or MRI, to exclude haemorrhagic stroke	Cancer outpatient
Potential time window for protective therapy	Per- or Post-conditioning	Per- or Post-conditioning	Pre-, Per- or Post-conditioning
Common co-morbidities and risk factors	Age, hypertension, hyperlipidaemia, diabetes, smoking	1/3 children and young adults [33], hypertension, hyperlipidaemia, diabetes, smoking	Age, female > male, dose, previous radiotherapy, Concurrent chemotherapy, underlying cardiac disease [14]
Common co-medication during treatment	P2Y_12_ inhibitor, aspirin, heparin	Tissue plasminogen activator (tPA)	Chemotherapeutic agent/s
Clinically available treatment for preventing cell death	Reperfusion therapy by PCI or CABG	Reperfusion therapy by thrombolysis ± mechanical thrombectomy	Dexrazoxane^a^
Primary outcome	MACE (Major Adverse Cardiac Events)	Modified Rankin score at 90 days (Neurological function)	Acutely: LV dysfunction. Chronically: progression to heart failure, death
Blood biomarkers of injury	Troponin	None clinically available	Troponin, persistently elevated NT-proBNP [14]
Type of injury	Myocardial injury	Cerebral injury	Cancer + cardiac injury
Type of cellular injury	Ischaemia and reperfusion	Ischaemia and reperfusion	Cyto-toxic
Progression of ischaemia/reperfusion/toxicity injury	Majority of infarct occurs during early reperfusion, with gradual increase thereafter	Infarct increases gradually over several hours during ischaemia and reperfusion	Acute, “Early-onset toxicity” within 1 year, “late-onset toxicity” after 1 year [14]
Cause of deaths/disability	MI, cardiogenic shock, progression to heart failure	Deaths within the first few days are usually the direct consequence of brain damage from neurological complications^b^ [9]	Cancer, progression to heart failure

^a^Clinical use of dexrazoxane is limited by concerns of diminished anti-tumour efficacy

^b^Possible neurological complications include brain oedema, haemorrhagic transformation, seizures and epilepsy, recurrent stroke

Both ischaemic stroke and STEMI are usually caused by obstruction of a main conduit artery by a blood clot. In the heart, the clot typically forms in the region, where an atherosclerotic plaque has ruptured [99]. The resulting hypoperfusion in the ischaemic “area at risk” will lead to cell death if recanalization does not occur promptly. Some of the area at risk will be salvaged by reperfusion, and its effectiveness can be further improved by interventions such as ischaemic pre- or post-conditioning [31, 41, 54]. In the brain, thromboembolic stroke is more common, but up to 40% of all ischaemic strokes are of unknown aetiology [30]. In ischaemic stroke, there may be a zone of non-functioning but viable tissue that has the potential to recover its function if blood flow can be restored, for example, by therapeutic intervention. This region is referred to as the ischaemic “penumbra” [5].

At a cellular level, the response to ischaemia is broadly similar in the heart and brain [57]. Since neurons and cardiomyocytes rely on high rates of oxidative phosphorylation for the production of ATP, in the absence of oxygen, ATP is rapidly depleted. While cells can survive on ATP produced by glycolysis for a short time, eventually, this decreases to levels that are insufficient to maintain essential ion homeostasis, and Ca^2+^ begins to flood in and overload the cells. Reperfusion restores the essential flow of oxygen and nutrients to starved cells [21]. In both heart and brain, the mitochondria are the source of their own demise, as rapid re-activation of the electron transport chain results in a burst of superoxide production, which conspires with calcium to increase opening of the mitochondrial permeability transition pores (MPTP) [8, 28, 71, 89]. Above a critical threshold, damage is irreversible and catastrophic injury results in cell death, primarily by necrosis/oncosis. Other types of cell death such as necroptosis are also involved [69, 93]. Apoptosis is important in the brain, but its role in the reperfused heart is more controversial [17, 59, 62, 68]. Although the mechanisms of cellular injury caused by ischaemia–reperfusion are very similar in the heart and brain, the brain is uniquely sensitive to damage caused by glutamate released from depolarized cells which causes glutamate excitotoxicity [45, 83].

However, infarction causes more than just the death of cardiomyocytes or neurons. The vasculature is essential not just for delivery of oxygenated blood, but for insulating the parenchyma from blood constituents and excessive liquid. This is particularly important in the brain, where energy depletion and blood–brain-barrier dysfunction can result in malignant oedema, a major cause of death following stroke [9]. Disruption of the neurovascular unit (which comprises endothelial cells, pericytes, vascular smooth muscle cells, astrocytes, microglia, and neurons) may also lead to further neuronal death. The vessels of the heart have an analogous, non-fenestrated endothelial cell layer, which is in some sense a “blood-heart barrier”. Damage to the cardiac endothelium can also result in oedema [48].

The debate about the significance of the differences between brain and heart, and their impacts on protection, led to the question of what is the most important experimental outcome. In experimental myocardial infarction studies, the gold standard and primary measure of outcome is infarct size (as a percentage of area at risk) [15], which predicts progression to heart failure in patients [94]. However, in the brain, infarct location is far more important than infarct size per se in determining functional outcome. For this reason, both neuroscore and infarct size should be considered in neuroprotection studies. The use of multiparametric MRI to assess per-occlusion and follow-up brain damage has the potential to improve translation by providing the same imaging endpoints in both the pre-clinical and clinical settings [19]. One of the greatest fears for the neurologist ministering to a patient with ischaemic stroke is haemorrhagic transformation [9, 30], whereas in the heart, haemorrhage is not a prime concern.

**Fig. 1:**
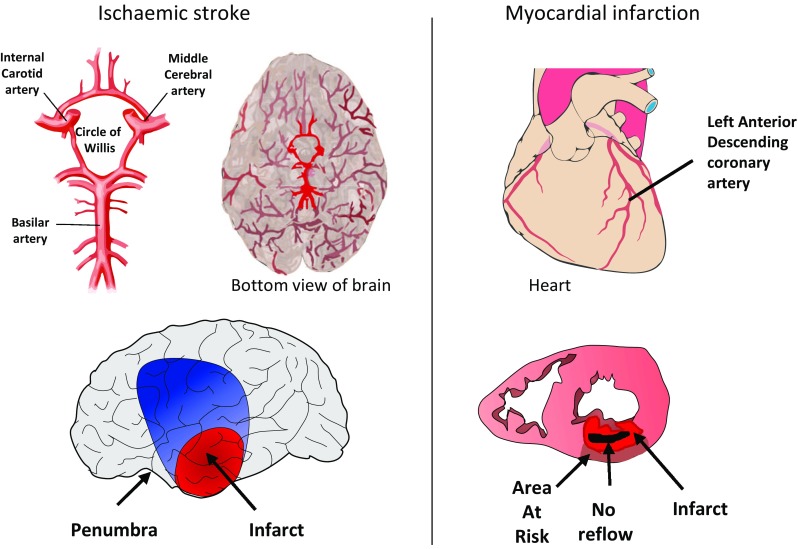
In the brain, middle cerebral artery occlusion results in a gradient of reduction in cerebral blood flow from the ischaemic core (red) through the penumbra and oligaemia (blue) to normally perfused cortex (grey). No reflow may also occur from 5 to 10 min. In the heart, occlusion of the LAD followed by reperfusion results in an ischaemia area risk in which a transmural infarct slowly develops, followed by the appearance of a zone of no reflow within the infarct

In both brain and heart, the degree of injury is highly dependent on the duration, extent and severity of ischaemia [51, 76]. The sole therapy available for each is reperfusion. The kinetics of reperfusion may be very different in patients treated with thrombolysis vs PCI or mechanical thrombectomy. Most experimental models, however, study reperfusion as an acute event. Rodent models of thromboembolic stroke amenable to thrombolysis do exist, but require more animals per group due to inherently greater experimental variability (see, for example, [102]). Reperfusion injury has been well studied in the heart, and is also thought to occur in the brain [4, 7, 68, 79, 83]. As such, targeting reperfusion injury should be considered an effective means of developing additional adjunctive therapies in patients with acute ischaemic stroke [62, 88]. Another key determinant of ischaemia–reperfusion injury in both heart and brain is the extent of collateralization. In the brain, some functional redundancy of blood supply is naturally provided by the circle of Willis (Fig. 1)—although the precise anatomy of these vessels can be quite variable between patients. Furthermore, the functionality of secondary collateral pathways such as leptomeningeal anastomoses is believed to be a main determinant of stroke outcome [39].

Even after successful recanalization of the occluded vessel, reperfusion at the level of the tissue may be limited—a situation called “no reflow”. No reflow occurs in both the heart and the brain but with very different kinetics and a partially distinct mechanism [48, 52, 64]. No reflow can occur within 5–10 min of ischaemia in the brain [3], and may, therefore, contribute to neuronal death, whereas in the heart it only occurs after 30 + min and its contribution to cell death is less clear [64]. The fact that the brain is confined within a rigid skull may contribute to the differences in the manifestation of no reflow [64]. In the brain, perfusion deficits occur in a gradient from the infarct through to an oligaemic region of mildly reduced blood flow, via an ischaemic penumbra of potentially salvageable tissue.

**Fig. 2:**
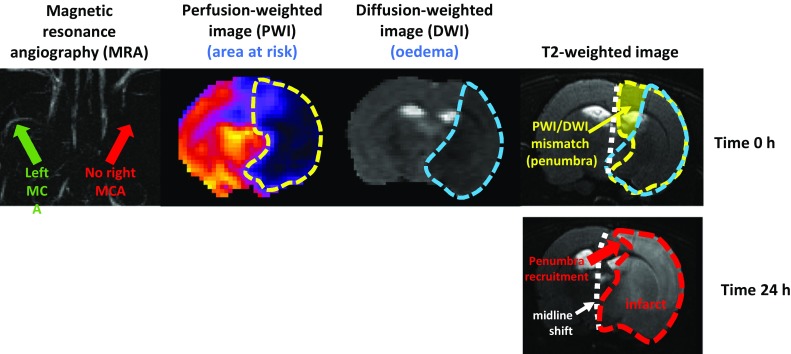
MRI images of a rat subject to middle cerebral artery (MCA) occlusion and reperfusion, illustrating recruitment of the ischaemic penumbra in the infarct. The top panels confirm the complete occlusion of the MCA and show the perfusion-weighted and diffusion-weighted images, which when combined reveal the ischaemic penumbra. In the lower panel, after 24 h part of the penumbra has been recruited into the area of infarct, and brain swelling has caused a quantifiable shift of the midline

With regard to the above discussion, it was unanimously agreed that experimentally; in both heart and brain, it is crucial to accurately determine the volume of tissue that is ischaemic and, therefore, at risk of infarction. While in the heart, this can be readily achieved by Evans blue staining ex vivo, or the use of microspheres, in the brain this is not a trivial matter due to its extensive collateralization. The clinical method of estimating the ischaemic penumbra by measuring the per-occlusion perfusion/diffusion mismatch by MRI can also be applied in animal models of transient stroke (Fig. 2) [29]. Thus, MRI has the potential to improve the overall methodology of pre-clinical neuroprotection studies, with the advantage that it can also be used to provide a measure of infarct size that matches well with tetrazolium chloride staining [20].

The use of rodent models in cardioprotection and neuroprotection has limitations. There are clearly major differences between lissencephalic brains of small mammals and gyrencephalic brains of larger species, which lead to the recommendation to confirm results in rabbits or non-human primates [66]. Similar reasoning is frequently used to support the need for cardioprotection studies in pigs [56] prior to clinical studies, although one might question whether healthy pig hearts, with their low native collateralization, are generally the ideal model of patients with coronary artery disease (CAD), who may or may not be highly collateralized.

One factor specific to MI, which may have contributed to the poor translation of cardioprotection between animal models of ischaemia-reperfusion injury and patients, is the clinical use of P2Y_12_ platelet inhibitors, which exhibit coagulation-independent cardioprotection in their own right [12, 23]. Although nearly all STEMI patients receive such medication, platelet inhibitors are not used at the time of recanalization in stroke for fear of causing haemorrhagic conversion. Thus, this potential confounding factor is only relevant to STEMI and not to ischaemic stroke.

At another level, several parallels can be drawn between the mechanism of cardiac injury caused by STEMI and anthracycline cardiotoxicity, including the role of oxidative stress, mitochondrial damage, and cardiomyocyte death [37, 38, 74]. It was noted that current clinical trials aim to treat heart failure after it has been detected, but not to prevent the cause—which is cardiomyocyte death. New experimental studies are needed of agents that are better able to protect the cardiomyocytes from anthracycline toxicity, with the added condition that they must absolutely not reduce the efficacy of the cancer treatment [74].

### Cyto-protection—the present situation

A number of issues were discussed at this workshop that are relevant to each of the three pathological scenarios described above. It was agreed that one of the most promising forms of cyto-protection is the phenomenon of remote ischaemic conditioning (RIC) [40, 46, 50]. RIC is a highly robust method of reducing myocardial infarct size in animal models [16] as well as in proof of concept clinical studies [51]. One outstanding issue that was discussed at the workshop relates to the RIC protocol, which remains to be optimized in humans. Despite efforts made in this regard in animal models [16, 60], clinical trials typically use a protocol of 3 or 4 cycles of 5 min upper limb ischaemia (maximum 200 mmHg) followed by 5 min reperfusion, which was effective in phase 1 trials [51]. Yet, no phase II trials have been performed.

RIC has repeatedly been shown to reduce the release of cell-death biomarkers such as troponin or creatine kinase in pilot studies of STEMI [51]. Interestingly, there are also indications of improvements in long-term clinical outcome with significant reductions in major adverse cardiac and cerebrovascular events (MACCE) [91] and mortality [36]. It was felt that STEMI remained the most important target for cardioprotection from RIC or pharmacological therapy. It was debated whether other settings such as CABG, which has had two neutral clinical outcome studies [42, 75], should still be considered amenable for cardioprotection [63, 96].

Results are eagerly awaited from the ERIC-PPCI/CONDI-2 study investigating the effect of RIC on clinical outcomes in patients presenting with an STEMI undergoing primary percutaneous coronary intervention [44]. All participants at the workshop agreed that the outcome of this pivotal study will determine the direction that cardiovascular research will take for the next decade. However, irrespective of the results of this study, what is true for the heart might not be true for the brain.

Evidence was shown that ischaemic preconditioning (IPC) may be able to protect cardiac cells from anthracycline toxicity, while not increasing the survival of cancer cells [73]. A clinical study is currently being undertaken to assess the efficacy of RIC in patients receiving doxorubicin for cancer therapy [22].

Derek Yellon presented data obtained using a new experimental rat model that could potentially be used to evaluate cardioprotection on a background of agents commonly administered to STEMI patients. These preliminary studies were designed to ascertain if it is possible to obtain an animal model more representative of patients and included the use of an anticoagulant, an analgesic and antiplatelet agent. The participants agreed that this represented a promising first step to overcoming some of the translational hurdles that have impaired translation.

The prevalence of obesity and diabetes is steadily increasing and is predicted to cause the incidence of myocardial infarction and strokes to soar in the next two decades. Diabetes and other co-morbidities worsen the outcome from both STEMI and ischaemic stroke, and also impair cardioprotective and neuroprotective strategies in animal models [32, 67, 80]. Given the above, Derek Yellon proposed that a multi-targeted strategy would be required to protect the heart or brain from IR injury as a way forward [25, 85].

However, despite the overall negative impact of diabetes on the cardiovascular system, it has perhaps been indirectly responsible for shining a glimmer of light onto the field of cardioprotection. There was an interesting discussion about the cardioprotective benefit that has now been seen in three, separate, large clinical trials of antidiabetic therapies, namely, the SGLT2 inhibitors [2, 101]. These results from recent large clinical studies show conclusively that cardioprotection is a viable option. What is now required is to ascertain the mechanism by which these agents elicit their protection [13].

### The way forward

A succinct set of ten guidelines were put forward at the previous Hatter meeting [12]. Many of these proposals have been incorporated into subsequent statements and recommendations. In particular, it was felt that, given the multiple redundancies in cell death pathways, targeting of a single pathway may be unable to afford sufficient protection for clinical benefit. As such, it is important to investigate all the forms of cell death to achieve maximum protection.

To increase the potency of protection and inhibit alternative death pathways, a multi-target therapeutic approach may be necessary to achieve a clinically meaningful benefit [25]. Additive cardioprotection has been seen, e.g., with the caspase 1 inhibitor VX-765 administered at reperfusion in P2Y_12_ receptor antagonist-treated rats [6]. Combination therapy may also have the potential to protect against acute ischaemic stroke, but this important concept remains unproven in this setting [81, 100]. To design a rational, multi-targeted approach, it is important to know the mechanisms and perform accurate dose–response experiments. One promising avenue of research is exosomes. These are nanoparticles that have been shown to be cardioprotective in animal models, which may be able to target multiple pathways via their protein and miRNA cargo [26, 27, 92, 98]. However, many questions remain, including the real identity of their cellular target in the heart, and optimal methods for their purification and delivery [27].

All participants recognized that a better methodology will be required in order to close the translational “gap”. Ultimately, two types of animal models are useful: one which is simplistic, conceptual and reductionist, which can inform about mechanisms and a second type of model that is complex, real-world, clinical, translational, and pragmatic, which can be used as a test bed towards clinical translation.

In line with the previous recommendations, a system is urgently needed to enable the conduct of multi-centre animal trials, much like the CAESAR network previously established in USA [61], or the MULTIPART network for neuroprotection [1]. Interestingly, such a multi-centre, blinded, randomized, controlled experimental infarct study was previously used successfully in the year 2000 to demonstrate that an adenosine A1 agonist at reperfusion was cardioprotective when administered prior to coronary occlusion in rabbits, but not when administered immediately prior to reperfusion [10]. All future studies should follow as closely as possible the appropriate guidelines [12, 15, 34, 65–67, 78] on effective translational research.
